# Paid Sick Leave and Risks of All-Cause and Cause-Specific Mortality among Adult Workers in the USA

**DOI:** 10.3390/ijerph14101247

**Published:** 2017-10-19

**Authors:** Daniel Kim

**Affiliations:** 1 Department of Health Sciences, Northeastern University, 360 Huntington Avenue, Robinson Hall, Suite 312C, Boston, MA 02115, USA; d.kim@northeastern.edu; 2 Department of Social and Behavioral Sciences, EHESP School of Public Health, Sorbonne Paris Cité, Paris Descartes University, 75006 Paris, France

**Keywords:** paid leave, social determinants, social factors influencing health, health inequity

## Abstract

*Background:* The USA is one of only a few advanced economies globally that does not guarantee its workers paid sick leave. While there are plausible reasons why paid sick leave may be linked to mortality, little is known empirically about this association. *Methods:* In a pooled USA nationally-representative longitudinal sample of 57,323 working adults aged 18–85 years from the National Health Interview Surveys 2000–2002, paid sick leave was examined as a predictor of all-cause and cause-specific mortality. Multivariate Cox proportional hazards models were used to estimate the impact of paid sick leave on mortality. *Results:* Having paid sick leave through one’s job was associated with 10% (hazards ratio, HR = 0.90; 95% CI = 0.81–0.996; *p* = 0.04), 14% (HR = 0.86; 95% CI = 0.74–0.99; *p* = 0.04), and 22% (HR = 0.78; 95% CI = 0.65–0.94; *p* = 0.01) significantly lower hazards of all-cause mortality after mean follow-up times of 11.1, 6.5, and 4.5 years, respectively. This study further identified associations of paid sick leave with 24% (HR = 0.76; 95% CI = 0.59–0.98; *p* = 0.03), and 35% (HR = 0.65; 95% CI = 0.44–0.95; *p* = 0.03) lower hazards of dying from heart diseases and unintentional injuries, respectively. *Conclusions:* To the author’s knowledge, this study provides the first empirical evidence on the linkages between paid sick leave and mortality and supports protective effects, particularly against heart diseases and unintentional injuries. The most salient association corresponded to a lag period of just less than five years. Social policies that mandate paid sick leave may help to reduce health inequities and alleviate the population burden of mortality among working adults in the USA.

## 1. Introduction

Growing evidence supports employment opportunities and policies as social determinants of health inequities and disease burden at the population level. Employment that does not offer paid sick leave is plausibly linked to worse health outcomes including a higher risk of mortality, including through reducing one’s earnings and job productivity. If one continues to work while sick, a phenomenon known as “sickness presenteeism” [1], the duration and severity of illness may also be lengthened, by not allowing for adequate medical attention and rest. In addition, paid sick leave may conceivably produce negative spillover effects—such as reducing the chances of spread of infectious diseases such as influenza to coworkers, and enabling care for sick children and elderly family members [2]. Empirical studies have also demonstrated an association between sick leave and lower earnings [3], and have linked sickness presenteeism to a higher risk of serious coronary events [1]. Furthermore, lack of paid sick leave has been shown to be a barrier to cancer screening and medical-care seeking in the United States of America (USA) [4]. To the author’s knowledge, however, no studies have yet explored the associations between paid sick leave and major health endpoints including mortality, signifying an important gap in the literature.

**Figure 1:**
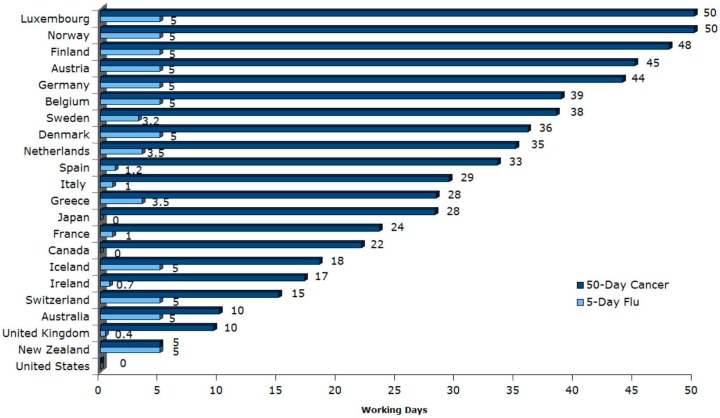
Paid Sick Days and Leave in 22 Countries, Median Worker. Source: [5].

Notably, the USA is one of only three advanced economies globally which does not guarantee its workers any paid sick leave for both short- and long-term illnesses [2,5]. The USA federal Fair Labor Standards Act (FLSA) does not require payment for time not worked due to sickness, and paid sick leave is a benefit based on an agreement between the employer and employee [6,7]. While in Canada decisions regarding short-term sick leave are made regionally at the provincial level, within the USA, no paid sick leave is mandated at the state level [2]. By contrast, with the exceptions of the USA, Canada, and Japan, 32 of 35 OECD countries including Australia, New Zealand, and countries in the European Union provide mandated support for both short- and long-term sick leave at the federal level (Figure 1) [2].

The argument for paid sick leave extends beyond health to human rights: According to the United Nations’ Universal Declaration of Human Rights, *“Everyone has the right to a standard of living adequate for the health and well-being of himself and of his family, including food, clothing, housing and medical care and necessary social services, and the right to security in the event of unemployment, sickness, disability, widowhood, old age or other lack of livelihood in circumstances beyond his control”* [8]. Like paid maternity leave, for which a similar health and human rights argument has been put forth and for which political and public support is rapidly growing in the USA [9,10,11], there is the potential for policy change in the USA to mandate paid sick leave for adult workers.

There are conceptual reasons for differential vulnerabilities to impacts of paid sick leave on mortality across subpopulations. For example, having more education may increase one’s knowledge and self-efficacy [12] to use sick leave to engage in positive health behaviors and obtain adequate medical care and rest. Moreover, associations may differ between men and women due to differences in gender roles (e.g., greater home and family responsibilities) [13], that modify the impacts of paid sick leave.

Using a nationally-representative cohort of adult male and female workers in the USA, the present study examined the associations between paid sick leave and all-cause and cause-specific mortality, to provide the first empirical evidence on this topic.

## 2. Materials and Methods

The National Health Interview Survey (NHIS) is an ongoing repeated cross-sectional survey of USA households, designed to be representative of the civilian, noninstitutionalized population [14]. NHIS consists of a core questionnaire that collects demographic, socioeconomic, psychosocial, lifestyle, and other health information for all family members in a sampled household and supplements that query information about specialized topics [14]. The final sample was constructed by pooling adult respondents from the 2000, 2001, and 2002 NHIS surveys between the ages of 18 and 85 who reported being employed in the previous week. These surveys had corresponding response rates of 72.1%, 73.8%, and 74.3% respectively.

The outcomes were all-cause and cause-specific mortality, based on the public-use NHIS Linked Mortality Files, with mortality follow-up data from the date of survey participation through 31 December 2011 [15]. Mortality status was ascertained through probabilistic record matching with the National Death Index (NDI). The public-use version of the NCHS Linked Mortality Files were subjected to data perturbation techniques which reduce the risk of participant re-identification and which have been shown to generate similar model results compared to the restricted-use version of the NCHS Linked Mortality Files [15,16]. All survey participants were included in the public-use Linked Mortality file regardless of linkage eligibility.

For the primary predictor variable, data was used on the presence of paid sick leave at their primary job, based on the survey item “*Do you have paid sick leave on this main job or business?*”

Model covariates consisted of baseline age, gender, educational attainment, income, history of any chronic conditions (heart disease, stroke, hypertension, diabetes, cancer, asthma, and chronic obstructive lung disease), and survey year. Race/ethnicity, marital status, family size, smoking, body mass index, type of occupation (according to Standard Occupation Classification Codes), number of employees at one’s job, number of years worked at one’s job, and receipt of welfare assistance were also considered as control variables in the models, but did not exhibit evidence of being confounders, and so were excluded from the models to maximize statistical power.

This study was approved by the Institutional Review Board at Northeastern University (IRB # 17-09-05).

### Statistical Analysis

Descriptive statistics were first calculated based on study participants included in the regression analyses. To examine the associations between the presence of paid sick leave at baseline and mortality, Cox proportional hazards models were then estimated, controlling for model covariates.

From these models, multivariate-adjusted hazard ratios were estimated for the associations between the presence of paid sick leave and the risk of mortality from specific underlying causes of death (heart diseases, I00–I09, I11, I13, I20–I51; cancer, C00–C97; chronic lower respiratory diseases, J40–J47; unintentional injuries, V01–X59, Y85–Y86; cerebrovascular diseases, I60–I69; Alzheimer’s disease, G30; diabetes mellitus, E10–E14; influenza and pneumonia, J09–J18; nephritis, nephrotic syndrome, and nephrosis, N00–N07, N17–N19, N25–N27; all other causes) according to the International Statistical Classification of Diseases, Injuries, and Causes of Death (ICD-10) guidelines and the risk of mortality from any cause [17]. Survival time was defined as the time from survey (the midpoint of the year quarter in which the interview was completed) of the survey year (2000, 2001, 2002) to the date of death (using the midpoint of the quarter in which the death occurred) or the end-date of follow-up (31 December 2011), whichever occurred first. Individuals who remained alive over the period of follow-up were treated as censored observations.

The presence of effect modification by age, sex, race/ethnicity, education, and income was respectively assessed using the Wald test to test the statistical significance of each added cross-product term for the corresponding interaction.

The proportional hazards assumption was tested by examining the significance of an added interaction term between paid sick leave and the follow-up time. Because this interaction term was statistically non-significant at the 5% level, the proportional hazards assumption was deemed to be met.

In sensitivity analyses, the robustness of the findings was explored after modifying the end-dates of follow-up to 31 December 2007 and 31 December 2005, and also re-estimating the models using logistic regression.

To control for sample design and non-response, all models incorporated survey weights. Because the pooled sample combined three survey years, each survey weight was calculated as the individual survey sample weight multiplied by one-third. All tests were two-tailed with a 5% significance level. All analyses were conducted using SAS Version 9.4 (SAS Institute, Cary, NC, USA).

## 3. Results

Table 1 shows the unweighted and weighted descriptive characteristics of the study samples analyzed in the Cox proportional hazards regression and logistic regression analyses. The weighted mean duration of follow-up was 132.8 months, or 11.1 years. After restricting the analytic sample to 60,918 individuals between the ages of 18 and 85, and removing those with missing values for mortality status (5.4%), the sample size was reduced to 57,323. The missing indicator method [18] was applied for missing observations on paid sick leave (1.6%), history of chronic conditions (1.1%), education (0.5%), and income (17.9%). In the weighted analytic sample, the mean age of 40.1 years, and 46.7% of the sample was female. 27.8% of the sample had attained at least a college education, and 56.2% reported having paid sick leave in their primary job. Nearly one-third of the sample (31.5%) had a past history of a chronic condition. In total, over the maximum nearly 12-year follow-up period, 2253 deaths occurred (cumulative incidence of mortality = 3.9%) (Table 1).

**Figure 2:**
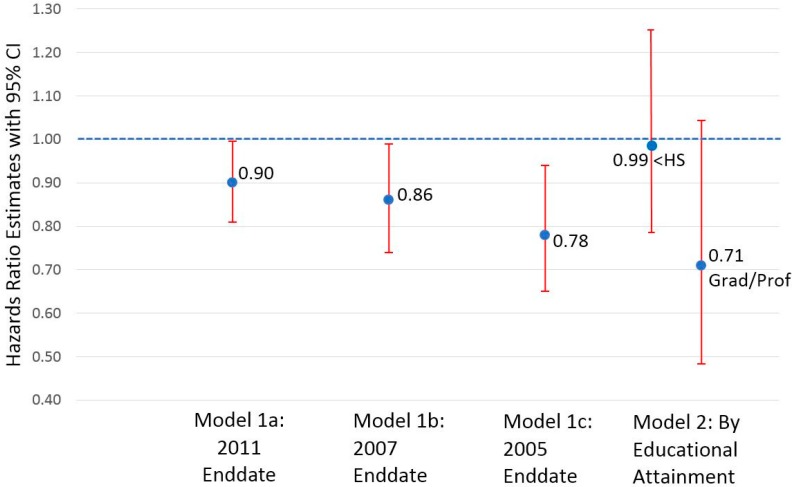
Hazard ratio estimates from Cox proportional hazards regression models for associations between paid sick leave and all-cause mortality by end-date of follow-up and by highest and lowest educational attainment, USA National Health Interview Surveys 2000–2002 (*n* = 57,323). All models adjusted for age, gender, educational attainment, income, history of a chronic condition (heart disease, stroke, hypertension, diabetes, cancer, asthma, or chronic obstructive lung disease) and survey year. Enddates of follow-up correspond to 31 December of the year noted. ‘<HS’ = less than high school educational attainment. ‘Grad/Prof’ = graduate or professional school educational attainment.

Figure 2 presents the multivariate-adjusted hazards ratio estimates from Cox proportional hazards regression models for the association between paid sick leave and all-cause mortality. Controlling for other factors, the presence of paid sick leave predicted a 10% lower hazards of all-cause mortality (Model 1a: hazards ratio, HR = 0.90; 95% CI = 0.81–0.996; *p* = 0.04). There was no evidence of effect modification by age, gender, or income (data not shown). By contrast, there was evidence of effect modification by education, with the highest (vs. lowest) educational attainment more strongly associated with all-cause mortality marginally non-significant at the 5% level (Model 2; HR = 0.71, 95% CI = 0.48–1.04, for those with graduate school or professional education vs. HR = 0.99, 95% CI = 0.79–1.24, for those with less than high school education; *p* = 0.051 for education interaction). When the enddates of follow-up were modified to 31 December 2007 and 31 December 2005 (with weighted mean durations of follow-up of 6.5 and 4.5 years, respectively), the presence of paid sick leave predicted 14% (Model 1b: HR = 0.86; 95% CI = 0.74–0.99; *p* = 0.04) and 22% (Model 1c: HR = 0.78; 95% CI = 0.65–0.94; *p* = 0.01) significantly lower hazards of all-cause mortality.

**Figure 3:**
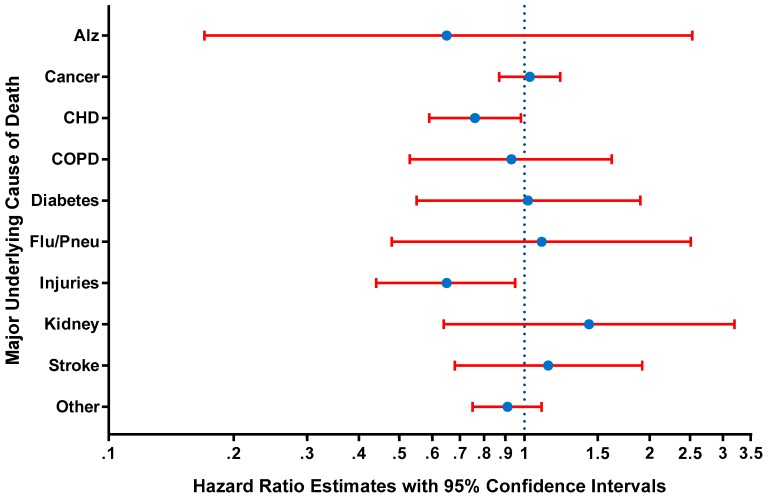
Hazard ratio estimates from Cox proportional hazards regression models for paid sick leave and mortality by major underlying cause of death, USA National Health Interview Surveys 2000–2002 (*n* = 57,323). All models adjusted for age, gender, educational attainment, income, history of a chronic condition (heart disease, stroke, hypertension, diabetes, cancer, asthma, or chronic obstructive lung disease) and survey year. ‘Alz’ = Alzheimer’s disease. ‘CHD’ = heart diseases. ‘COPD’ = chronic lower respiratory diseases. ‘Flu/Pneu’ = influenza and pneumonia. ‘Injuries’ = unintentional injuries. ‘Kidney’ = nephritis, nephrotic syndrome, and nephrosis. ‘Stroke’ = cerebrovascular diseases. ‘Other’ = all other causes.

Figure 3 shows the multivariate-adjusted hazard ratio estimates from Cox proportional hazards regression models for the associations between paid sick leave and mortality from specific underlying causes. Controlling for other factors, the presence (vs. absence) of paid sick leave significantly predicted a 24% lower hazards of death from heart diseases (HR = 0.76; 95% CI = 0.59–0.98; *p* = 0.03), and a 35% lower hazards of death from unintentional injuries (HR = 0.65; 95% CI = 0.44–0.95; *p* = 0.03). Associations for other underlying causes of death were not statistically significant at the 5% level (Figure 3). For kidney diseases, a stronger association was observed in women than men (*p* = 0.046 for gender interaction), while for the category of other causes of death, the association was stronger in men than women (*p* = 0.03 for gender interaction). There was no evidence of effect modification by age, education, or income (data not shown).

In additional sensitivity analyses, the principal findings for the significant associations between paid sick leave and cause-specific and all-cause mortality remained robust when logistic regression was instead used (all-cause mortality: odds ratio, OR = 0.89; 95% CI = 0.80–0.99; *p* = 0.03; mortality from heart diseases: OR = 0.76; 95% CI = 0.59–0.99; *p* = 0.04; mortality from unintentional injuries: OR = 0.65; 95% CI = 0.44–0.96; *p* = 0.03).

## 4. Discussion

In this study based on a nationally-representative sample of working men and women in the USA aged 18–85 years, associations were found between paid sick leave and mortality. Having paid sick leave at one’s job was associated with 10%, 14%, and 22% significantly lower risks of all-cause mortality after average follow-up times of 11.1, 6.5, and 4.5 years, respectively. This study further identified associations of paid sick leave with 24% and 35% lower risks of dying from heart diseases and unintentional injuries, respectively. Evidence of a stronger protective association in those with the highest versus lowest level of education was also determined.

This study possessed several major strengths, including its reliance on a nationally-representative sample of working-aged adults, thereby strengthening the generalizability of our findings within the USA; its use of longitudinal data, which allowed for temporality to support causation; and its employment of linkages to the National Death Index, which reduced the presence of selection bias due to attrition. The additional assessment of effect modification enabled the identification of any subgroup differences in associations by age, gender, and socioeconomic status. Finally, this study conducted sensitivity analyses including modeling shorter latency periods based on earlier enddates of follow-up, and using logistic regression to examine the robustness of the findings to alternative model specification.

Previous empirical studies have found associations that are compatible with the findings in the current study. For instance, Asfaw et al. [19] demonstrated that those with paid sick leave versus those without paid leave were 28% less likely (OR = 0.72, 95% CI = 0.53–0.99) to develop non-fatal occupational injuries, and had a 25% lower risk of experiencing job separation, an event which can plausibly impact future mortality risk [20]. Furthermore, those lacking paid sick leave have been shown to be three times more likely (OR = 3.1, 95% CI = 2.1–4.8) to forgo medical care for themselves [21], and to have a 32% lower odds (OR = 0.68, 95% CI = 0.50–0.91) of using emergency department services on a frequent basis [22]. Conversely, other studies have identified significant associations between paid sick leave and positive health-related outcomes including influenza vaccinations [23], cancer screening [4], and use of preventive health services [24]. All of these health outcomes are conceivably related to mortality, and collectively, these studies lend plausibility to the observed association between paid sick leave and all-cause mortality. The association with non-fatal occupational injuries in the study by Asfaw et al. [19] is also consistent with the relation with mortality from unintentional injuries observed in the current study.

This study found evidence for effect modification by educational attainment, with stronger associations between paid sick leave and all-cause mortality among those with graduate or professional education compared to those with less than high school education. Conceivably, those who attained the highest level of education may have been more likely to hold stable jobs with consistent paid sick leave policies over the study follow-up period. They may then have benefited more strongly from the protective effects of paid sick leave against mortality. A previous study showed that self-efficacy plays a substantial role in explaining the lower risk of frailty in older adults with a high (versus low) educational level [12]. Likewise, individuals with graduate or professional education may possess greater self-efficacy to employ their sick leave time to seek medical care, obtain appropriate rest, and engage in positive health behaviors, which in turn may confer them with stronger long-term protection against mortality compared to those with less than high school education. Moreover, analogous to the education differences observed in the current study, a past study determined that the association between psychosocial work demands and sickness absence and presenteeism was larger in countries higher on the Human Development Index, an index for which average education level is a principal constituent indicator [25].

In sensitivity analyses, the association between paid sick leave and all-cause mortality was strongest with a mean follow-up period of 4.5 years. This temporally-specific association plausibly reflects the average time that it takes for paid sick leave to adversely affect mortality. Likewise, previous studies have identified temporally-specific lag periods such as between metropolitan statistical area (MSA)-level income inequality and individual-level cognitive function [26] and between air pollution and cardiovascular outcomes [27]. In the present study, the observed associations spanning a relatively short term may possibly signify short-term impacts in existing vulnerable populations such as those with co-morbid conditions, who are already at higher risk of mortality.

Based upon the results of the current study, a federal mandate that expands paid sick leave to all adult workers—with approximately 70.2% of the population aged 20-64 being employed [28] and an estimated 43% and 11% of those in the private and public sectors lacking paid leave, respectively [2,29]—could have sizeable direct population health benefits. Notably, the anticipated overall benefits would likely exceed the direct benefits, as the policy-relevant impacts of lack of paid sick leave include increased morbidity of illnesses, lost productivity, and negative spillover health effects such as forgoing medical care for family members [21] or the increased spread of influenza in the workplace [30]. In addition, there is growing public support for expanding paid sick leave to all workers—with a 2017 poll by the Pew Research Center finding that 85% of Americans support a mandate for workers to receive paid sick leave [11]. Implementation of mandated paid sick leave in local jurisdictions including Seattle [31] and San Francisco [32] has been further shown to be successful according to several metrics including increases in paid sick leave offering and strong support by employees for the sick leave policy changes [29,30]. Together, the expected significant public health impacts and apparent public support for paid sick leave favors it as a feasible option by policymakers. While making such legislative changes at a federal level would demand more political will than at the municipal or state levels, the potential public health benefits that could be reaped are much larger in scale.

Several limitations of this study should be noted. First, while multiple factors were considered and/or controlled, residual confounding is still possible. Second, the information gathered on paid sick leave was self-reported for the availability of paid sick leave, not the number of days actually taken, and did not differentiate between paid sick days and extended paid sick leave i.e., for illnesses lasting several weeks. It is possible that the health effects could differ according to these different types of paid sick leave or the number of paid sick days taken. Third, the public-use file that was employed did not permit disaggregation of underlying causes of death beyond the 10 categories provided. As a result, more salient associations for certain causes of death may have been obscured. Fourth, while we captured paid sick leave for employment at baseline, individuals may have moved jobs after baseline with possible changes in paid sick leave policies, which in turn could influence the risk of mortality. Such information if collected in future studies would help to further elucidate the associations observed. Fifth, despite the relatively high individual year survey response rates ranging from 72.1% to 74.3%, there is still some potential for non-response bias. Last, only the direct health effects on individuals were considered in this study. Indirect benefits including positive spillover health effects of paid sick leave stemming from reducing the spread of infectious diseases [2] and permitting care for family members [21,33] are additional considerations that would provide valuable inputs for decision-making by policymakers at the federal level.

## 5. Conclusions

In summary, to the author’s knowledge, this study provides the first published evidence on the linkages between the presence of paid sick leave and mortality, and identifies significant protective associations, particularly for deaths from heart diseases and unintentional injuries. The most salient association corresponded to a lag period of just less than five years. Because mortality including from heart diseases and unintentional injuries disproportionately affects minority and lower socioeconomic groups [34,35], reducing mortality burden should have the overall impact of reducing health disparities. Over the long term, federal social policies that mandate paid sick leave in the USA may help to reduce health inequities and alleviate the population burden of mortality among working adults in the USA.

## Figures and Tables

**Table 1:** Descriptive characteristics of unweighted and weighted analytic samples, USA National Health Interview Surveys 2000–2002.

	Unweighted Sample (*n* = 57,323)	Weighted Sample (*n* = 133,408,667)
Mean follow-up (mos.)	132.9 (17.1)	132.8
Mean age (years)	40.5 (12.8)	40.1
Gender		
Male	28,222 (49.2%)	53.3%
Female	29,101 (50.8%)	46.7%
Education		
<High school	7709 (13.4%)	11.7%
High school/GED	16,086 (28.1%)	29.0%
Some College	17,772 (31.0%)	31.0%
College	10,166 (17.7%)	18.3%
Graduate School	5314 (9.3%)	9.5%
Missing	276 (0.5%)	0.5%
Income		
$0–9999	2682 (4.7%)	3.2%
$10,000–19,999	5251 (9.2%)	6.2%
$20,000–34,999	9772 (17.0%)	13.8%
$35,000–54,999	10,928 (19.1%)	18.5%
$55,000–74,999	7460 (13.0%)	14.8%
$75,000+	10,967 (19.1%)	24.7%
Missing	10,263 (17.9%)	18.8%
History of Chronic Conditions		
Yes	18,239 (31.8%)	31.5%
No	38,480 (67.1%)	67.5%
Missing	604 (1.1%)	1.0%
Paid sick leave		
Yes	32,180 (56.1%)	56.2%
No	24,233 (42.3%)	42.3%
Missing	910 (1.6%)	1.5%
Death prior to 31 December 2011		
Yes	2253 (3.9%)	3.6%
No	55,070 (96.1%)	96.4%

For the unweighted sample, the standard deviations for continuous variables are shown in parentheses. For categorical variables, the corresponding proportions are shown in parentheses.
